# Non‐native species have multiple abundance–impact curves

**DOI:** 10.1002/ece3.6364

**Published:** 2020-06-04

**Authors:** David L. Strayer

**Affiliations:** ^1^ Cary Institute of Ecosystem Studies Millbrook NY USA; ^2^ Graham Sustainability Institute University of Michigan Ann Arbor MI USA

**Keywords:** biological invasions, bivalves, density‐impact function, *Dreissena*, impacts, invasive species, management, space‐for‐time substitution

## Abstract

The abundance–impact curve is helpful for understanding and managing the impacts of non‐native species. Abundance–impact curves can have a wide range of shapes (e.g., linear, threshold, sigmoid), each with its own implications for scientific understanding and management. Sometimes, the abundance–impact curve has been viewed as a property of the species, with a single curve for a species. I argue that the abundance–impact curve is determined jointly by a non‐native species and the ecosystem it invades, so that a species may have multiple abundance–impact curves. Models of the impacts of the invasive mussel *Dreissena* show how a single species can have multiple, noninterchangeable abundance–impact curves. To the extent that ecosystem characteristics determine the abundance–impact curve, abundance–impact curves based on horizontal designs (space‐for‐time substitution) may be misleading and should be used with great caution, it at all. It is important for scientists and managers to correctly specify the abundance–impact curve when considering the impacts of non‐native species. Diverting attention from the invading species to the invaded ecosystem, and especially to the interaction between species and ecosystem, could improve our understanding of how non‐native species affect ecosystems and reduce uncertainty around the effects of management of populations of non‐native species.

## Graphical abstract

The abundance–impact curve is a useful tool for understanding and managing the impacts of invasive species. Using models based on the impacts of the zebra mussel, I show that a single invasive species can have radically different abundance–impact curves in different habitats. This means that managers must be careful to use the correct abundance–impact curve and that scientists should avoid using space‐for‐time substitution to understand the impacts of invaders.

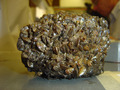

## 1 INTRODUCTION

Non‐native species are of concern because of their impacts. Whether the invader affects biodiversity, ecosystem function and services, human economies, or human health (e.g., Blackburn et al., 2014; Gallardo, Clavero, Sánchez, & Vilà, 2016; Lockwood, Hoopes, & Marchetti, 2013; Ricciardi, Hoopes, Marchetti, & Lockwood, 2013), it is the impacts of the invader, rather than the invader itself, that usually is the primary concern. Despite the central importance of impacts, many useful contributions about the impacts of specific invaders (e.g., Higgins & Vander Zanden, 2010; Vilà et al., 2011), and some general frameworks and empirical studies that apply broadly across taxa (e.g., Blackburn et al., 2014; Crystal‐Ornelas & Lockwood, 2020; Dick et al., 2014; Parker et al., 1999; Pearse, Sofaer, Zaya, & Spyreas, 2019), we are far from having satisfactory understanding or predictive power about the impacts of non‐native species (e.g., Crystal‐Ornelas & Lockwood, 2020; Ricciardi et al., 2013; Strayer, Solomon, Findlay, & Rosi, 2019).

**FIGURE 1:**
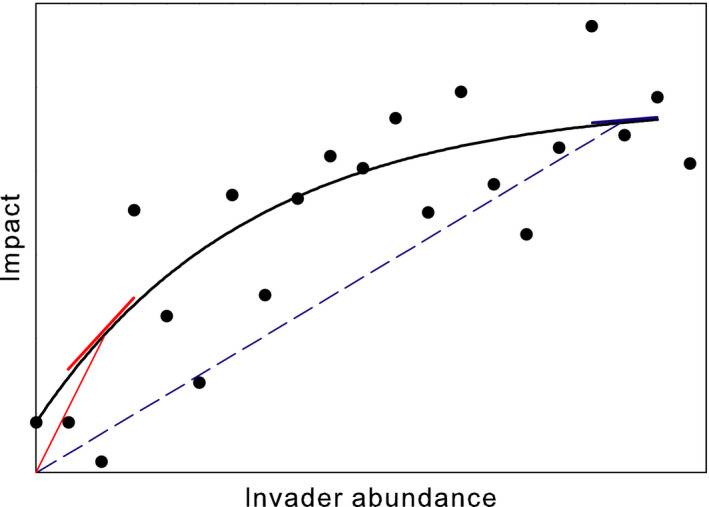
A hypothetical abundance–impact curve (black curve, based on black data points), which shows the total impact of a population of an invader as a function of its abundance. The slopes of the red and blue lines show average (dashed lines) and marginal (solid lines) per capita effects at two values of invader abundance

One useful general approach that links the invader with its impacts is the abundance–impact curve (Figure 1) (=density‐impact function [DIF]; Norbury, Pech, Byrom, & Innes, 2015), in which some measure of the abundance (e.g., population density, biomass) of a non‐native species is plotted against some measure of its total impact (e.g., Sofaer, Jarnevich, & Pearse, 2018; Yokomizo, Possingham, Thomas, & Buckley, 2009). The abundance–impact curve represents a substantial advance over earlier approaches (e.g., Parker et al., 1999) because it accommodates nonlinear relationships between abundance and impact, in which the marginal per capita effect can vary with invader abundance. It therefore identifies a critical distinction between the average and marginal per capita effects of an invader. The shape and parameters of this curve are highly relevant to management, because they allow managers to estimate the expected benefits of reducing the population of the invader by a given amount, which can be weighed against the expected costs of that reduction (e.g., Sofaer et al., 2018; Yokomizo et al., 2009). Especially in the last decade, scientists have published abundance–impact curves of problematic invaders (e.g., Benkwitt, 2015; Strayer, Solomon, et al., 2019; Thiele, Kollmann, Markussen, & Otte, 2010), as well as broad empirical analyses of the impacts of non‐native species that are based on abundance–impact curves (e.g., Bradley et al., 2019; Norbury et al., 2015; Pearse et al., 2019). These studies have provided insights into the basic ecology of species invasions, as well as information that could be useful to managers.

A potential problem with impact theories in general, and with abundance–impact curves in particular, is that impacts have been regarded chiefly as a property of the invading species (e.g., Ricciardi et al., 2013; Sofaer et al., 2018). Thus, it is common to see reference to *the* abundance–impact curve of a species, as if each species had a single abundance–impact curve. If the invaded ecosystem has been considered at all, it has been included implicitly (e.g., in the per capita effect term of Parker et al.’s [1999] equation), or treated as a secondary modulator of impacts. I argue here that the invading species and the invaded ecosystem are partners in determining impact and that both must be considered explicitly in effective theories of impacts. Furthermore, once we include the invaded ecosystem, we see that there generally will not be a single abundance–impact curve for a species, but multiple, noninterchangeable abundance–impact curves, each of which applies over limited domains (types of ecosystems, types of invaders, types of impacts). I will explore these ideas using simple models of the expected impacts of *Dreissena* (zebra and quagga mussels), ecologically and economically important invaders that have been well studied (e.g., Crystal‐Ornelas & Lockwood, 2020; Gallardo et al., 2016; Higgins & Vander Zanden, 2010).

## 2 ABUNDANCE–IMPACT CURVES OF *DREISSENA*: TWO EXAMPLES

**FIGURE 2:**
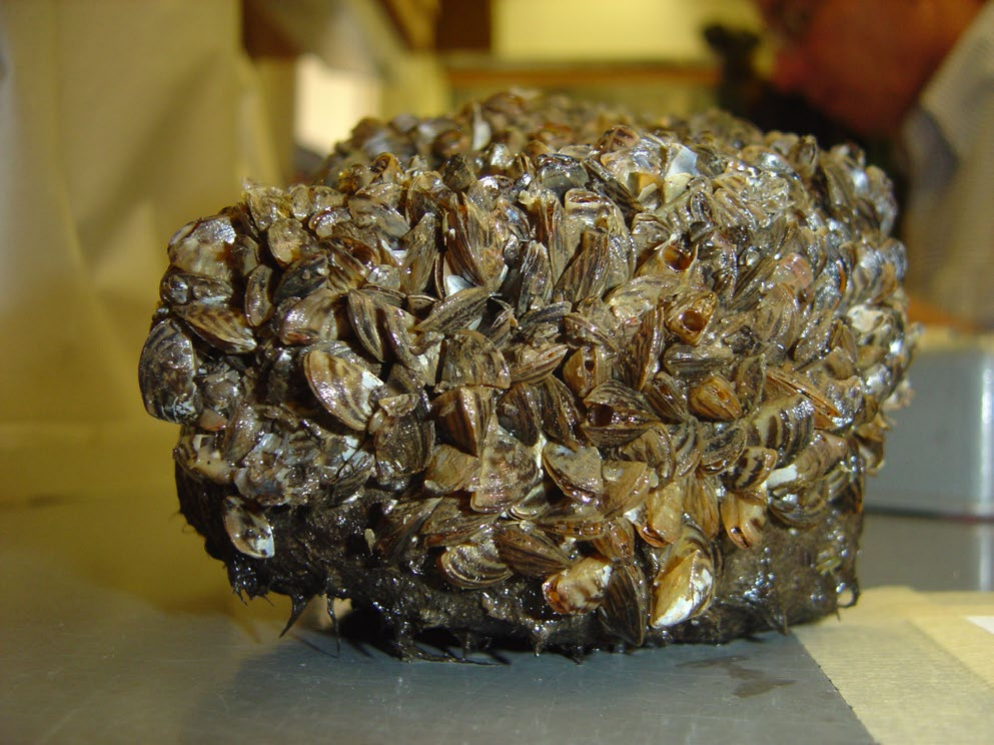
Zebra mussels, *Dreissena polymorpha*, covering a rock taken from the bottom of the Hudson River. Photograph by Heather Malcom

*Dreissena* species (Figure 2) are native to the Ponto‐Caspian region of southeastern Europe and southwestern Asia. Since the early 19th century, they have been spread widely through Western Europe and North America, chiefly through commercial shipping and recreational boating (Benson et al., 2019; van der Velde, Rajagopal, & bij de Vaate, 2010). They often form dense populations and have large ecological and economic impacts (summarized by Connelly, O'Neill, Knuth, & Brown, 2007; Higgins & Vander Zanden, 2010; Ricciardi, 2003; Strayer, 2009) as a result of their suspension feeding, shell building, and fouling. *Dreissena* has impacts that are broadly similar to many other species of freshwater, estuarine, and coastal marine bivalves that have been spread widely around the world by humans (e.g., *Corbicula*, *Limnoperna*, *Mytilopsis*, *Rangia*, and various species of oysters and mussels), and so represents an important class of invaders.

### 2.1 Example 1: shell accumulation

*Dreissena* plays many roles in ecosystems (e.g., Higgins & Vander Zanden, 2010; Ricciardi, 2003); here, I will here explore two roles that are simple and well understood enough to analyze with simple, quantitative models. The first is the accumulation of empty shells and shell fragments (“shell hash”) on the sediments. These empty shells change the surface roughness, texture, porosity, permeability, and chemistry of sediments, thereby affecting habitat for benthic animals, interstitial biogeochemistry, near‐bottom hydrodynamics, and exchanges of materials between the water and sediments (Gutiérrez, Jones, Strayer, & Iribarne, 2003; Ricciardi, 2003). Shell production by *Dreissena* and other mollusks can be large, approaching rates of wood production (in terms of mass) in temperate forests (Gutiérrez et al., 2003).

The amount of shell hash that accumulates on sediments depends on the rate at which empty shells are produced by dying animals and the rate at which they are dissolved, buried, or washed downstream by the ecosystem. For simplicity, I assume that burial and export are negligible, so that the dynamics of shell hash are determined by production and dissolution, as follows:

$$\frac{dS}{dt}=M-kS$$

where *S* is the standing stock of shell hash, *M* is the quantity of shell material entering the spent shell pool through mortality of living animals, and *k* is the instantaneous loss rate of spent shells. At steady state, mortality is equal to the production of spent shells (*P*) and (d*S*/d*t* = 0), so the quantity of shell hash will be *P*/*k*, where *k* depends on water chemistry and currents (Strayer & Malcom, 2007).

I will model shell accumulation in three ecosystems: a hardwater lake in which shell dissolution is slow (*k* = −0.05/year; rates estimated from Strayer & Malcom, 2007), a moderately hardwater lake in which shell dissolution is moderately fast (*k* = −0.3/year), and moderately hardwater river in which shell dissolution is fast (*k* = −2/year). I chose these three systems because they cover most of the range of conditions under which dense populations of *Dreissena* occur (Whittier, Ringold, Herlihy, & Pearson, 2008). (*Dreissena* does live in waters supersaturated in calcium carbonate, where even smaller absolute values of *k* would be expected, but not in very soft waters, where shell dissolution would very fast [*k* < −2/year].)

**FIGURE 3:**
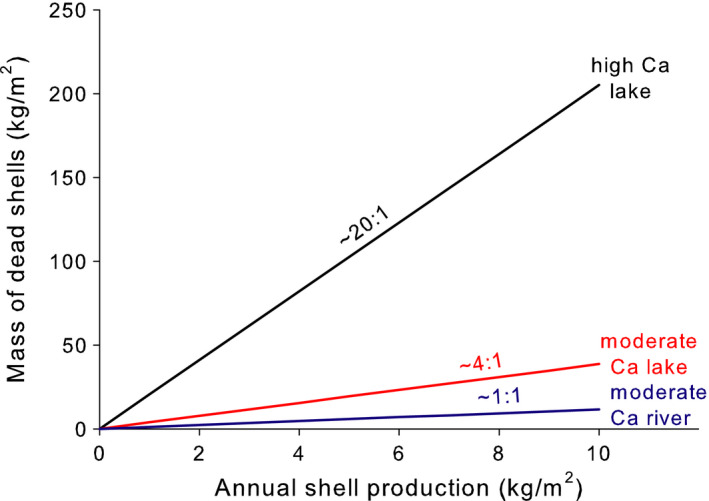
The amount of dead shell material that would accumulate (at equilibrium) across a range of constant *Dreissena* population sizes (expressed as shell production rates) in three model ecosystems (black line = hardwater lake, red line = moderately hardwater lake, blue line = moderately hardwater river; see text for details). The ratios shown above the lines are the ratio of equilibrial shell accumulation to annual production

I begin by considering the amount of shell hash that would accumulate, at equilibrium, by *Dreissena* populations of different sizes in each of these three hypothetical ecosystems. Again, the range of *Dreissena* population sizes used roughly matches the range expected in nature (Strayer & Malcom, 2007); note that population size is expressed here as the rate of shell production. In this first scenario, the amount of shell hash that accumulates on the sediments depends strongly on both the size of the *Dreissena* population and the characteristics of the ecosystem, to a roughly equal extent (Figure 3). In this example, the abundance–impact curve is always simple and of the same form (linear) across different ecosystems, and the difference across ecosystems is easily understood and modeled as a simple difference in slopes. The slopes depend on the shell dissolution rate, which can be estimated roughly from water chemistry and movement, or more precisely from simple litter‐bag studies (Strayer & Malcom, 2007). Furthermore, because shell dissolution rates are a function of shell size and thickness (Ilarri, Sousa, Amorim, & Sousa, 2019; Strayer & Malcom, 2007), it would be possible to extend this simple framework to cover other species of shell producers.

**FIGURE 4:**
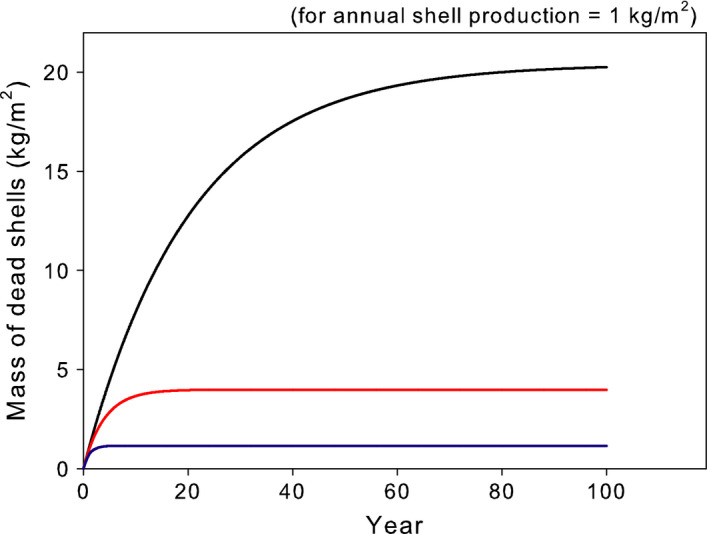
The amount of dead shell accumulated in three model ecosystems over time, assuming a constant shell production rate of 1 kg/m^2^ year. Black line = hardwater lake, red line = moderately hardwater lake, blue line = moderately hardwater river

However, the impacts of shell accumulation are cumulative, not instantaneous, so this example has interesting temporal dynamics, which also depend on the characteristics of the ecosystem. I will now relax the assumption of steady state and model the temporal dynamics of shell accumulation in different ecosystems. In this second scenario, I assume a constant *Dreissena* population and calculate the time course of shell accumulation in the three model ecosystems (Figure 4). As we already saw, the equilibrial amount of shell hash (the asymptotes in Figure 4) differs among ecosystems. In addition, the rate at which that asymptote is approached differs among ecosystems; systems with high dissolution rates approach equilibrium rapidly (within ~5 years), whereas systems with low dissolution rates take several decades to reach equilibrium. Thus, the ecosystem affects the dynamics of impacts as well as their long‐term equilibria.

But of course *Dreissena* populations vary over time; in many cases, year‐to‐year variation is approximately an order of magnitude (Strayer, Adamovich, et al., 2019). I next model the temporal dynamics of shell hash accumulation in different ecosystems that support temporally variable populations of *Dreissena*. Temporal variability of shell production in these populations mimics the year‐to‐year variation in *Dreissena* biomass in the Hudson River, a population with moderately high interannual variation (Strayer, Adamovich, et al., 2019). For simplicity, I modeled accumulation of shell hash only for the ecosystems with the highest and lowest rates of shell dissolution (i.e., the hardwater lake and the moderately hardwater river).

**FIGURE 5:**
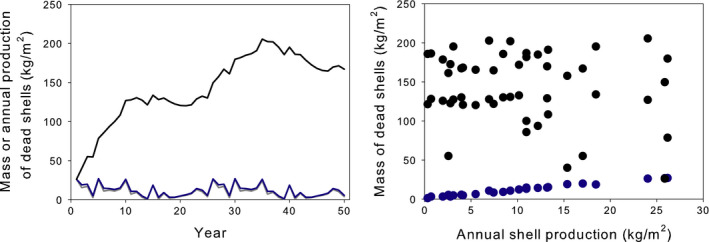
Left. Temporal dynamics of shell production (thin gray line, nearly obscured by blue line) and shell accumulation in two model ecosystems (black line = hardwater lake, blue line = moderately hardwater river). Right. Relationship between measured annual shell production and current shell accumulation in each year of study, for two model ecosystems (black circles = hardwater lake, blue circles = moderately hardwater river)

In the river with high dissolution rates, shell accumulation equilibrates rapidly with shell production, shell accumulation closely tracks shell production (Figure 5, left), and impact measured in any year is still a clear linear function of current *Dreissena* population size (Figure 5, right). However, when rates of shell dissolution are lower, the ecosystem equilibrates slowly with inputs, shell accumulation is not closely coupled with instantaneous rates of shell production (Figure 5, left), and there is no apparent relationship between the current impact and *Dreissena* population size (Figure 5, right). For a cumulative impact such as shell accumulation, impact at any time *t* will be a weighted function of invader population size over some temporal window preceding that time. Because the ecosystem determines the dynamics of the impact, the width of that window and the appropriate weighting function are determined by the characteristics of the ecosystem and will differ across ecosystems.

### 2.2 Example 2: provision of macrophyte habitat

The second example of *Dreissena* impact is the increase in the area of the photic zone available for colonization by submersed macrophytes. *Dreissena* typically increases water clarity by removing phytoplankton and other particles from the water column (Higgins & Vander Zanden, 2010; Higgins, Vander Zanden, Joppa, & Vadeboncouer, 2011). This can increase the area of lake or river bottom colonized by rooted plants and benthic algae (Zhu, Fitzgerald, Mayer, Rudstam, & Mills, 2006), which in turn can have large and far‐reaching effects on the food web, provision of habitat for fish and invertebrates, and biogeochemical processes and exchanges between the sediment and water column (Carpenter & Lodge, 1986; Jeppesen, Søndergaard, Søndergaard, & Christoffersen, 1998).

**FIGURE 6:**
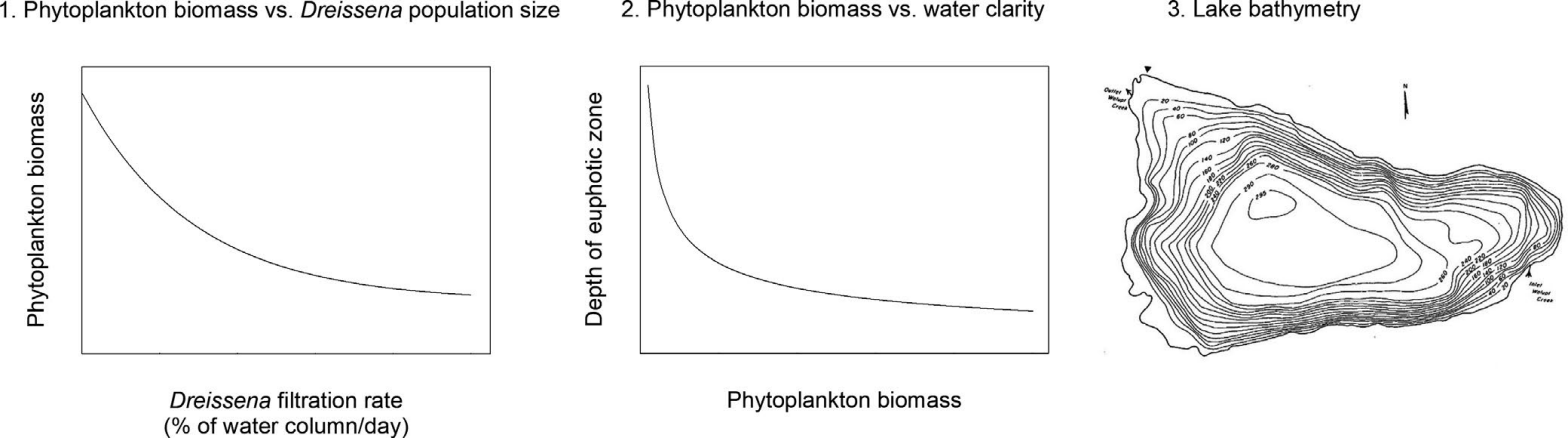
The three pieces of information needed to estimate the relationship between *Dreissena* population size and the area available for colonization by submersed macrophytes

Three pieces of information are needed to connect *Dreissena* abundance with the increase in area available for submersed macrophytes (Figure 6): (i) the relationship between *Dreissena* abundance (now expressed as aggregate filtration rate of the population) and phytoplankton biomass (as concentration of chlorophyll *a*); (ii) the relationship between phytoplankton biomass and water clarity; and (iii) the bathymetric map (technically the hypsographic curve) of a body of water. The relationship between *Dreissena* population filtration rate and phytoplankton biomass is known only approximately. For the purposes of this exercise, I assumed that phytoplankton biomass declines exponentially with *Dreissena* filtration rate as (Figure 6, left) follows:

$${\text{chl}}_{\text{post}}=0.2{\text{chl}}_{\text{pre}}+0.8{\text{chl}}_{\text{pre}}e^{\left(-0.0347\text{DFR}\right)}$$

where chl_pre_ and chl_post_ are the chlorophyll concentrations before and after the *Dreissena* invasion and *DFR* is the *Dreissena* filtration rate (as % of the water column/day). This equation is consistent with previous analyses and data (Caraco, Cole, & Strayer, 2006; Higgins & Vander Zanden, 2010; Strayer, Solomon, et al., 2019). The relationship between phytoplankton biomass and water clarity was well explored in the classical eutrophication literature; I used the relationship of Rast and Lee (1978) and shown in Figure 6 (center):

$$\log_{10}\text{Secchi}\;\text{depth}=-0.473\log_{10}\text{chl}+0.803$$

where Secchi depth is in m and chlorophyll (chl) is in µg/L. For bathymetry, I will use three contrasting model lakes: (i) a conical basin with a maximum depth of 5 m (“shallow”); (ii) a conical basin with a maximum depth of 50 m (“deep”); (iii) a lake of intermediate depth (maximum = 15 m), but with a pronounced shelf between 2.5 m and 3 m (“shelf”; such shelves are common in lakes). I ran this model for an unproductive lake (preinvasion chlorophyll concentration of 3 µg/L) and a productive lake (preinvasion chlorophyll concentration of 30 µg/L). I further assumed that the light extinction coefficient (*η*) was equal to the Secchi depth/1.7 (Wetzel, 2001) and that submersed macrophytes could survive to the depth reached by 5% of surface light (Moss, 2010).

**FIGURE 7:**
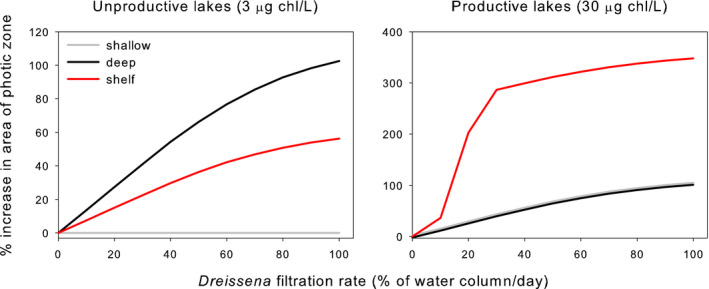
Expected increase in area of lake bottom suitable for submersed vegetation, as a function of *Dreissena* population size in different ecosystems. Black lines = deep, conical lake basin, gray lines = shallow, conical lake basin, red line = lake basin with a shelf. Note the difference in *y*‐axis scaling between the two panels. In the right panel, the black and gray lines have been shifted slightly for visibility (they actually lie on top of one another)

This model produced several notable results (Figure 7). In some ecosystems, the relationship between *Dreissena* population size and area available for submersed macrophytes was positive and asymptotic, simply with differences in slope among the different lakes. However, other ecosystem types showed fundamentally different relationships. For the shallow, unproductive lake, the arrival of *Dreissena* had no effect on the area available for submersed macrophytes, regardless of the density of *Dreissena*, because the entire lake bottom was well lighted enough for submersed macrophytes before *Dreissena* arrived. The abundance–impact curve for the productive “shelf” lake was highly nonlinear, with steep increases in macrophyte habitat at *Dreissena* filtration rates of 10%–30% of the water column/day contrasting with much lower rates over other parts of the range. Such idiosyncratic responses would occur in the many lakes that have nonlinear hypsographic curves (i.e., nonconical basins).

This second example again shows that impacts are a joint property of the *Dreissena* population and the ecosystem and that a wide range of abundance–impact curves are possible (both in terms of parameters and shapes). Despite this complication, impacts are predictable if we explicitly consider both the invader and the ecosystem. As in the first example, it seems likely that this analysis could be extended to accommodate the activities of other non‐native species in the same functional group as *Dreissena* (suspension feeders in this second example), if we express their population sizes in terms of filtration rates. In contrast to the shell accumulation example, the impacts here are more or less instantaneous (the light environment should closely follow changes in filtration rates, even though there may be some lags in the responses of macrophytes), so that the history of the invasion is less likely to be critical.

These two examples show that the characteristics of the ecosystem can be fundamentally important in defining the abundance–impact curve and must be explicitly considered if we hope to understand that curve. As others (e.g., Pearse et al., 2019; Ricciardi, 2003; Ricciardi et al., 2013) have noted, there are different curves for different impacts of a single invader (i.e., shell accumulation vs. water clarification). These differences may be especially marked between instantaneous and slow, cumulative impacts. Furthermore, very different attributes of the ecosystem are important for these different impacts—water chemistry and movement for shell accumulation versus lake bathymetry and productivity for provision of submersed macrophyte habitat. Likewise, the abundance of the invader may best be expressed in different ways (e.g., population density, biomass, shell production rate, filtration rate) depending on the impact being considered.

## 3 IMPLICATIONS OF ECOSYSTEM SENSITIVITY FOR HORIZONTAL STUDIES (SPACE‐FOR‐TIME SUBSTITUTION) IN INVASION ECOLOGY

Up until now, I have not been explicit about what the points in the abundance–impact curve (Figure 1) represent. In fact, there are at least three distinct versions of the abundance–impact curve, depending on what the points represent. These three versions will generally not be interchangeable in terms of their shapes, parameters, or applications. All three curves have the abundance of the invader on the *x*‐axis and one of its impacts on the *y*‐axis (as in Figure 1). In the first formulation (“within system”), the points on the graph come from a single ecosystem. This could be either a single ecosystem in nature sampled over different times, each with a different abundance of the invader, or experimentally manipulated to produce different abundances, or from an experiment using different abundances of the non‐native species in replicates of the same ecosystem. In the second formulation (“cross‐system snapshot”), the points are snapshots, each representing a single sample from different ecosystems. In the third formulation (“cross‐system, long‐term”), the points are long‐term means from different ecosystems.

**FIGURE 8:**
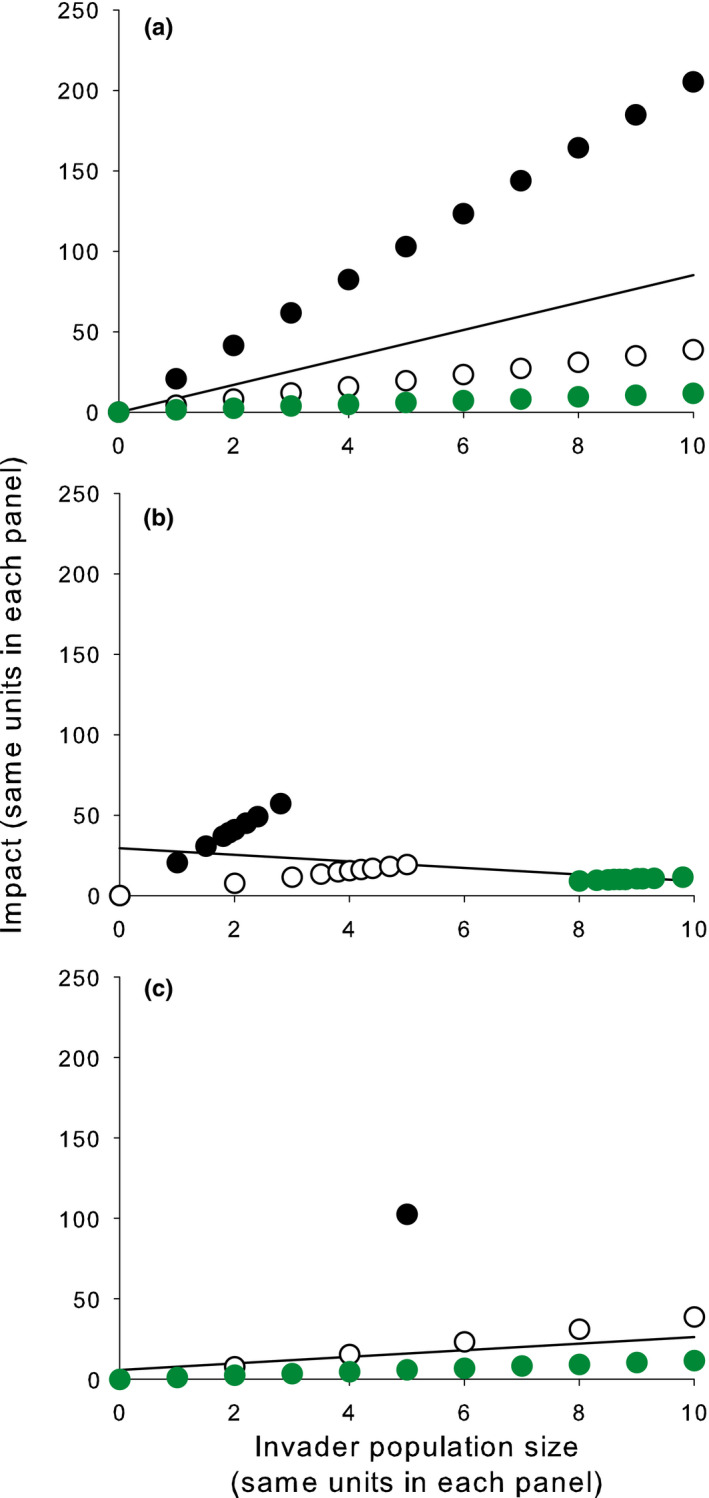
Expected impact of an invader in populations of lakes (each point is a lake; different colors represent lakes with different linear within‐system abundance–impact curves; black circles = high‐slope ecosystems, white circles = moderate slope ecosystems, green circles = low‐slope ecosystems) in different landscapes (see text for further explanation) (a) equal numbers of each type of lake, invader densities evenly spaced; (b) equal numbers of each type of lake, invader densities unevenly spaced; (c) unequal numbers of each type of lake, invader densities evenly spaced. The black regression lines are abundance–impact curves fitted through the entire collection of points

To see the difference among these three abundance–impact curves, consider a very simple example in which within‐system impacts are noncumulative, linear on invader abundance, but with different slopes in different types of ecosystems. Further assume that invader abundance varies over time in each ecosystem and that different landscapes hold three types of ecosystems (with a high slope, moderate slope, and low slope, respectively, to their abundance–impact curves) in different proportions. Snapshot samples taken from such a landscape will produce data points whose distribution depends on (a) the within‐system abundance–impact curves; (b) the distribution of invader densities over time within each ecosystem; and (c) the proportion of each kind of ecosystem in the landscape (and possibly (d) the proportion of each kind of ecosystem in the sample, if the ecosystems are not sampled using a representative sampling design). The three selected examples in Figure 8 show that highly varied distributions of points, and therefore highly varied abundance–impact curves, can be produced from snapshot samples taken from a single simple system. It does not take much imagination to see that almost any distribution of data points and any shape of abundance–impact curve can be obtained from cross‐system snapshot sampling, even if the system has a very simple underlying structure, if different ecosystems have different abundance–impact curves. This problem becomes even more severe if the system has a more complex underlying structure (e.g., abundance–impact curves that are nonlinear or different in shape in different ecosystems, cumulative impacts). Except in the case of coincidence, the abundance–impact curves obtained by snapshot sampling (the black lines in Figure 8) will generally not match any of the within‐system abundance–impact curves in shape, parameters, or even sign. Specifically, the fitted lines will not accurately predict the results of changing invader abundances in any ecosystem in the landscape and can even (as in Figure 8b) produce predictions of the wrong sign.

If we sample the ecosystems in this simple example repeatedly to get their long‐term mean abundances and impacts, we will obtain less noisy versions of plots like those shown in Figure 8. If the abundance of the invader does not vary much over time, the long‐term curve will closely resemble the snapshot curve, whereas if invader abundance within ecosystems varies greatly over time, the long‐term curve will look a lot cleaner than the snapshot version. However, neither cross‐system curve will generally resemble the within‐system curves, in either shape or parameters.

If the within‐system abundance–impact curve is nonlinear (which will often be the case; e.g., Benkwitt, 2015; Norbury et al., 2015; Strayer, Solomon, et al., 2019), the snapshot and long‐term cross‐system curves will also differ from one another in shape and parameters. They will differ because the mean value of a dependent variable evaluated at a series of points along a nonlinear function is not the same as the value of the dependent variable evaluated at the mean value of the independent variable (cf. Karamata's Inequality or Jensen's Inequality—Denny, 2017). This problem can range in severity from negligibly small to large depending on the degree of nonlinearity in the within‐system abundance–impact curves and the amount of temporal variation in invader abundance.

Thus, even if the impact of the invader is not a cumulative function of invader abundance, the three different abundance–impact curves are equivalent only under special conditions. The within‐system curve and the snapshot curve will be the same only if invader impact is unaffected by the characteristics of the ecosystem (i.e., if a single abundance–impact curve applies to all ecosystems in the sample). The snapshot curve and the long‐term cross‐system curve will be the same only if all within‐system invader‐impact curves are linear. And all three curves will be the same only if both of these conditions apply—if the abundance–impact curve is linear and identical in all ecosystems in the sample. These conditions seem unlikely to apply to many impacts of invaders.

Cumulative impacts introduce additional complications. We have seen that if we wish to obtain an interpretable within‐system abundance–impact curve for cumulative impacts, we must consider (and weight) invader abundance over some window of time, and both the width of the time window and the weighting function can differ across ecosystems. Consequently, even if abundance–impact curves are similar across all ecosystems, the snapshot approach will not produce interpretable results for cumulative impacts, unless the impact equilibrates rapidly (e.g., the blue line in Figure 5, left) or the invader's abundance is stable over time. Nor will the long‐term cross‐system abundance–impact curves equal the within‐system curves, even if abundance–impact curves are similar across all ecosystems (although they may be less erroneous than the snapshot results), because the temporal weighting functions will generally be nonlinear. This further restricts the conditions under which cross‐system and within‐system abundance–impact curves will resemble one another.

Some of the problems with horizontal designs can be solved by careful matching of study sites, so that differences in a relevant ecosystem characteristic are minimized (i.e., so that the study ecosystems all fall along a single abundance–impact curve, as do points of the same color in Figure 8), or by explicitly including the relevant ecosystem characteristics in the abundance–impact model. Indeed, both of these strategies have been recommended or used in horizontal studies of impacts (e.g., Jackson, Ruiz‐Navarro, & Britton, 2015; Pyšek et al., 2012; Staska, Essl, & Samimi, 2014; Thiele et al., 2010). Nevertheless, such strategies may fail to produce reliable abundance–impact curves if the sites are poorly matched, the within‐system abundance–impact curve is nonlinear, or impacts are cumulative. All of these problems are likely to be common. Furthermore, because the different impacts of a single species may be sensitive to different ecosystem characteristics (as in the two *Dreissena* examples), a set of study sites that is well‐matched for studying one impact may be ill‐suited to study another impact of the same species.

## 4 WHY DOES THIS MATTER?

It has been well appreciated that ecosystem characteristics help to determine the establishment, spread, and local abundance of non‐native species (e.g., Leung & Mandrak, 2007; Lewis et al., 2017; Lockwood et al., 2013). The examples presented here emphasize that ecosystem characteristics can also strongly affect the abundance–impact curve. That is, ecosystems help to set not only the occurrence and abundance of a non‐native species at a site, but also its per capita effects.

Abundance–impact curves can be important to several important scientific and management problems (e.g., Sofaer et al., 2018; Thiele et al., 2010; Yokomizo et al., 2009). Most obviously, an accurate abundance–impact curve can help managers evaluate the benefits and costs of proposed management actions to reduce the abundance of a non‐native species (Yokomizo et al., 2009). Abundance–impact curves are essential to schemes to assess the regional impacts of non‐native species (e.g., Thiele et al., 2010; Vander Zanden, Hansen, & Latzka, 2017). They can also provide a standardized way by which to compare impacts of one non‐native to another, or natives to non‐natives (Pearse et al., 2019). Using the wrong parameters and shape for an abundance–impact curve can therefore have serious consequences for scientific understanding, and incur unnecessary monetary and environmental costs from inappropriate management actions (e.g., Yokomizo et al., 2009).

I have shown here that within‐ and across‐system abundance–impact curves can be radically different in shape and parameters (Figure 8). Nevertheless, previous studies have generally failed to recognize the existence of different kinds of abundance–impact curves, regarded them as interchangeable (e.g., Sofaer et al., 2018), used across‐system curves to judge how the impacts of a non‐native species would change if its abundance was to change (e.g., Bradley et al., 2019), or mixed different kinds of abundance–impact curves (e.g., Norbury et al., 2015). Such uncritical use of abundance–impact curves is likely to cause confusion and slow scientific progress, lead to misleading understanding of the impacts of non‐native species, cost money (Yokomizo et al., 2009), and damage ecosystems that are subject to inappropriate management actions.

## 5 THE WAY FORWARD

Ecosystems can strongly influence abundance–impact curves of non‐native species, complicating their use and interpretation. The examples presented here for *Dreissena*, which are relatively realistic, show that the ecosystem is of first‐order importance, roughly as important as *Dreissena* abundance, in determining two selected impacts (shell accumulation and provision of macrophyte habitat). There has been little systematic examination of how other per capita impacts of *Dreissena* vary across ecosystems, but the information that is available suggests that these impacts do vary substantially across different kinds of ecosystems. Thus, apart from any effect of *Dreissena* abundance, Caraco et al. (1997), Higgins et al. (2011), and Sarnelle, White, Horst, and Hamilton (2012) found that impacts on phytoplankton depend on epilimnetic volume, stratification, turbidity, and nutrient content; Strayer, Hattala, and Kahnle (2004, figure 8 and associated text) suggested that impacts on fish communities depend greatly on system morphometry, hydrology, and turbidity, as well as the species composition of the fish community; impacts on native bivalves may depend on hydrodynamics and sediment type (Strayer & Malcom, 2018; Zanatta et al., 2015); and Strayer, Caraco, Cole, Findlay, and Pace (1999, figure 9) found large differences in many attributes of ecosystems that were invaded by *Dreissena* populations of similar density. It therefore seems likely that many impacts of *Dreissena* depend substantially on ecosystem characteristics and cannot be reduced to a single abundance–impact curve.

These conclusions about *Dreissena* probably apply to other non‐native species. Many of the impacts of non‐native species may depend on the characteristics of the invaded ecosystem, in addition to the abundance of the invader, and the list of relevant ecosystem characteristics must be diverse, depending on the impact being considered. For instance, the impacts of a nitrogen‐fixing plant or a nitrogen‐recycling animal must depend on whether the ecosystem is strongly nitrogen limited or nitrogen replete (e.g., Atkinson, Capps, Rugenski, & Vanni, 2017; Luo et al., 2014; Scherer‐Lorenzen, Venterink, & Buschmann, 2007). More generally, we can expect impacts of non‐native species to depend on factors such as the structure of the food web (e.g., Vander Zanden, Olden, Thorne, & Mandrak, 2004), whether the ecosystem is rich or poor in nutrients (as for nitrogen), productive or unproductive (as in the second *Dreissena* example), highly retentive or rapidly flushed (e.g., Lucas & Thompson, 2012), stable or highly disturbed, highly heterogeneous or relatively uniform (e.g., Lucas, Cloern, Thompson, Stacey, & Koseff, 2016; MacRae & Jackson, 2001), to name a few obvious possibilities. Therefore, for many invaders, it will be more useful to think of multiple abundance–impact curves, each applying to a defined range of impacts, functional groups of species, and types of ecosystems, and each with its own scientific and management applications, rather than a single curve.

Furthermore, although this essay has focused on non‐native species, it should be obvious that these considerations apply equally to native species, and so have broad application in ecology. Ecologists and managers often consider trying to increase the abundance of a native species to increase the ecosystem services it provides (e.g., Coen et al., 2007; Kreeger, Gatenby, & Bergstrom, 2018) or reduce the abundance of a native species to reduce its harmful impacts (e.g., Beguin, Tremblay, Thiffault, Pothier, & Côté, 2016). Abundance–impact curves can help to predict the likely changes in impacts resulting from a projected change in abundance and thus assess the costs and benefits of management actions. As for non‐native species, it will be essential in such applications to correctly choose and parameterize the abundance–impact curve.

But although it seems clear that ecosystems *can* strongly influence the abundance–impact curve, surely there must also be many cases in which the influence of the ecosystem is small enough to ignore, especially if the domain of study systems is carefully defined. But how often *do* ecosystems matter? Can we identify the conditions under which ecosystems are most likely to matter? Clearly, we need better theoretical and empirical explorations of how (and how much) ecosystems affect abundance–impact curves. In many cases, we know enough about the mechanisms of impact that we should be able to predict what characteristics of an ecosystem ought to affect a specified impact (as in the *Dreissena* examples), and use models, experiments, or field observations to assess the importance of ecosystem characteristics to invader impacts. It may eventually be possible to develop a theoretical or empirical basis for separating the situations in which impacts are sensitive to ecosystem characteristics from those in which impacts are robust to variation in ecosystems.

How should we proceed in the interim until we satisfactorily understand the importance of ecosystem characteristics to abundance–impact curves? If scientific studies show that the ecosystem has little or no influence on the abundance–impact curve, then a single abundance–impact curve can be applied for a given impact of a non‐native species across sites, and any of several methods can be used to estimate the abundance–impact curve (keeping in mind the caveats about cumulative impacts discussed above). However, to the extent that the impacts of non‐native species do depend on the characteristics of the invaded ecosystem as well as those of the invader, any satisfactory understanding of invader impacts will have to explicitly consider ecosystems as well as species. This means that we will need to gather and analyze data separately for each kind of ecosystem (cf. Norbury et al., 2015; Thiele et al., 2010) or include ecosystem characteristics in general models of impacts (e.g., Pyšek et al., 2012), limit extrapolations to well defined domains (of impact type, species functional group, and ecosystem type; Norbury et al., 2015), and take care to apply the correct kind of abundance–impact curve to each application. In particular, unless until ecosystems are shown to have little influence on a given impact, abundance–impact curves derived from cross‐system designs should be viewed skeptically and used very cautiously. Likewise, if abundance–impact curves are to be used for management, it will be important to consider whether such curves are reliable and have been based on sound science. But to make an obvious point, management of non‐native species is based on considerations other than abundance–impact curves, as valuable as they may be, so there is no reason to postpone management of a non‐native species until reliable abundance–impact curves become available.

The problems raised in this essay will complicate analyses of abundance–impact curves and non‐native species impacts. However, addressing these problems should improve our understanding of how non‐native species affect ecosystems and reduce uncertainty around the effects of management of populations of non‐native species. Furthermore, as the *Dreissena* examples suggest, these are likely to be tractable problems and can be solved if invasion ecologists divert some of their attention from the invading species to the invaded ecosystem, and especially to the interaction between species and ecosystem.

## CONFLICT OF INTEREST

None declared.

## AUTHOR CONTRIBUTION

**David L. Strayer:** Conceptualization (equal); Data curation (equal); Formal analysis (equal); Funding acquisition (equal); Investigation (equal); Methodology (equal); Project administration (equal); Resources (equal); Supervision (equal); Validation (equal); Visualization (equal); Writing‐original draft (equal); Writing‐review & editing (equal).

## Data Availability

No new data were collected for this study. Results of simulation models are freely available at https://doi.org/10.25390/caryinstitute.11367455.
